# Tight junctions at the blood brain barrier: physiological architecture and disease-associated dysregulation

**DOI:** 10.1186/2045-8118-9-23

**Published:** 2012-11-09

**Authors:** Anny-Claude Luissint, Cédric Artus, Fabienne Glacial, Kayathiri Ganeshamoorthy, Pierre-Olivier Couraud

**Affiliations:** 1 INSERM U1016, Institut Cochin, Paris, France; 2 CNRS, UMR 8104, Paris, France; 3 Université Paris Descartes, Sorbonne Paris Cité, Paris, France

**Keywords:** Blood–brain barrier, Tight junction, Neurovascular unit, Kinases, Signaling pathways

## Abstract

The Blood–brain barrier (BBB), present at the level of the endothelium of cerebral blood vessels, selectively restricts the blood-to-brain paracellular diffusion of compounds; it is mandatory for cerebral homeostasis and proper neuronal function. The barrier properties of these specialized endothelial cells notably depend on tight junctions (TJs) between adjacent cells: TJs are dynamic structures consisting of a number of transmembrane and membrane-associated cytoplasmic proteins, which are assembled in a multimolecular complex and acting as a platform for intracellular signaling. Although the structural composition of these complexes has been well described in the recent years, our knowledge about their functional regulation still remains fragmentary. Importantly, pericytes, embedded in the vascular basement membrane, and perivascular microglial cells, astrocytes and neurons contribute to the regulation of endothelial TJs and BBB function, altogether constituting the so-called neurovascular unit.

The present review summarizes our current understanding of the structure and functional regulation of endothelial TJs at the BBB. Accumulating evidence points to a correlation between BBB dysfunction, alteration of TJ complexes and progression of a variety of CNS diseases, such as stroke, multiple sclerosis and brain tumors, as well as neurodegenerative diseases like Parkinson’s and Alzheimer’s diseases. Understanding how TJ integrity is controlled may thus help improve drug delivery across the BBB and the design of therapeutic strategies for neurological disorders.

## Review

### Background

The BBB maintains the homeostasis of the central nervous system (CNS) by (i) strictly limiting the passive diffusion of polar substances from the blood to the brain, (ii) mediating the transport of nutrients to the brain parenchyma as well as the efflux from the brain of toxic metabolites and xenobiotics, (iii) regulating the migration of circulating immune cells [1-3]. Formed by specialized vascular endothelial cells, the BBB is tightly controlled by pericytes, embedded in the vascular basement membrane, perivascular microglial cells, astrocytes and neurons which altogether constitute the neurovascular unit (NVU), a concept highlighting the functional cell-cell interactions supporting BBB function.

BBB endothelial cells display a unique phenotype characterized by the presence of TJs and the expression of specific polarized transport systems. TJs constitute the most apical intercellular junctional complex in polarized epithelium and endothelium, with three key biological functions: a barrier to paracellular diffusion of blood-borne polar substances [4], a fence preventing the lateral diffusion of lipids and integral membrane proteins, thus maintaining cell polarization [5-7] and an intracellular signaling platform which will be described below.

Brain endothelial TJ strands, like epithelial TJs, are composed of integral membrane proteins (occludin, claudins and junctional adhesion molecules (JAMs)) involved in intercellular contacts and interactions with cytoplasmic scaffolding proteins such as zonula occludens (ZO) proteins, actin cytoskeleton and associated proteins, such as protein kinases, small GTPases [8] and heterotrimeric G-proteins [9].

Excellent reviews have recently been published on the architecture of TJ complexes in epithelial and brain endothelial cells [10,11]. Here we will briefly recall the main features of the structural organization of TJs at the BBB and will focus on transcriptional regulation, post-translational modifications and subcellular localization of TJ proteins and their consequences for BBB integrity with exposure to various environmental stimuli and during CNS disorders.

## Components of TJs in brain endothelial cells

As in polarized epithelial cells where TJs have been mostly studied, the TJ backbone in brain endothelial cells consists of transmembrane proteins (occludin, claudins and JAMs) which recruit a number of membrane-associated cytoplasmic proteins.

### Transmembrane proteins as the BBB TJ backbone

Occludin (60kDa), a tetraspan integral membrane protein, was the first TJ-specific protein identified [12,13] in epithelial cells and shown to be functionally important for barrier function [14]. It is a member of the family of TJ-associated marvel proteins (TAMP) with tricellulin (marvelD2) [15] and marvelD3 [16,17]. Both the MARVEL transmembrane domain of occludin, encompassing the four transmembrane helices, and its coiled coil cytosolic C-terminus were recently described to mediate its lateral (i.e. *cis*-) oligomerization in epithelial MDCK cells [18-20]. More precisely, cystein residues in these domains are directly involved in oligomerization through disulfide bridge formation. This process being redox-sensitive, oligomerization of occludin likely contributes to the redox-dependency of the TJ assembly [20,21]: whereas normoxia conditions support occludin oligomerization and contribute to TJ assembly, oxidative stress associated with hypoxia-reoxygenation [22] or inflammation [23,24] results in TJ disruption. This novel concept that occludin plays a key role in the redox regulation of TJs has been very recently reviewed [25].

In addition, the second extracellular domain of occludin is required for its stable assembly in TJs [26]. Indeed, synthetic peptides corresponding to this domain were shown to perturb TJ permeability barrier in epithelial cells [27-29]. The important contribution of occludin to TJ function is illustrated by the observations that ectopic expression of chicken occludin induced the formation of TJ-like structures in Sf9 insect cells [30], while increasing electrical resistance in MDCK cells [31]. Conversely, occludin degradation induced by viruses or bacteria (like HIV-1 Tat protein or *Neisseria meningitidis*), is associated with increased permeability in primary or immortalized human brain microvascular endothelial cells, respectively [32,33]. However, well-developed TJ strands were reported in cells lacking occludin (human or guinea pig testis) [34] and between adjacent occludin-deficient epithelial cells [34,35]; together with the report that occludin deficient-mice are viable, exhibiting normal TJs morphology as well as intestinal epithelium barrier function, these observations indicate that occludin is dispensable for TJ formation [36,37].

Claudins constitute a large family of 20-27kDa membrane proteins (with four transmembrane domains) expressed in TJs in various cell types [4,38-40] (endothelial and epithelial cells). Brain endothelial cells predominantly express claudin-3 and claudin-5 [41,42], claudin-12 likely being also expressed [43,44]. A large corpus of data clearly establishes the key contribution of claudin-3 and claudin-5 to TJ formation and integrity at the BBB. Indeed, exogenous expression of claudin-5 strengthens barrier properties in cultured rat brain endothelial cells [44], whereas depletion of claudin-5 induces the disruption of the BBB in genetically-altered mice [43] and in cultured human brain endothelial cells [9]. Claudins support TJ integrity via their capacity of cis- and trans-homodimerization as well as heterodimerization, notably through their second extracellular loop, as recently reported for claudin-5 [45-47]. Claudin-5 can interact with claudin-3 [48,49] and the selective loss of the latter during autoimmune encephalomyelitis or human glioblastoma is associated with BBB breakdown [41].

Beside occludin and claudins, JAMs, although not essential to TJ formation in epithelial and endothelial cells, may be involved in the facilitation of assembly of TJ components and in the establishment of cell polarity by recruiting the polarity complex (Par-3/Par-6/aPKC: see below) to TJs [50,51].

### Membrane-associated cytoplasmic proteins in BBB TJs

A number of cytoplasmic proteins have been described to associate with TJ transmembrane proteins and to contribute somehow to TJ integrity in epithelial and brain endothelial cells. Among them, the PDZ domain-containing, membrane-associated guanylate kinase (MAGUK) family members have been largely documented: zonula occludens-1 (ZO-1, 225kDa) [52], ZO-2 (160kDa) [53], and ZO-3 (130kDa) [54]. ZO-1 forms heterodimers with ZO-2 and ZO-3 [54-56]. ZO proteins interact with the C-terminal domain of claudins via their first PDZ domain (PDZ1) [57], to JAMs by the third PDZ domain (PDZ3) [58] and to occludin via their GUK domains [55,56,59]. It is well established that ZO proteins are essential to the assembly of claudins [60], occludin [35] and JAM-A [61] at TJs, then anchoring this multimolecular complex to the actin cytoskeleton [62]. Par-3 (also known as ASIP) [63] binds to JAM proteins [64-66] and recruits to TJs atypical protein kinase C [67] and Par-6 [68], the three proteins then forming a Planar Cell Polarity (PCP) complex in polarized epithelial cells [69]. Only very recently was their expression confirmed also in brain endothelial cells [70].

Among additional TJ-associated proteins, heterotrimeric G-proteins (Gαi) were first described, in association with ZO-1, to contribute to TJ biogenesis and maintenance in epithelial and brain endothelial cells [71-73]. Gαi2 proteins were reported to be involved in T-lymphocyte extravasation, including in brain [74,75]. More recently, we reported that Gαi2 interacts with claudin-5 and that its depletion increases brain endothelial cell permeability *in vitro* and delays TJ reassembly after hyperosmotic shock (induced by a high concentration mannitol treatment) [9]. On the basis of these observations, we proposed that claudin-5 and Gαi2, whether they interact directly or indirectly, might control TJ integrity as components of a multiprotein complex, including caveolin, ZO-1 linked to the actin cytoskeleton and possibly also, occludin and MUPP-1.

## Physiological regulation of TJ assembly by the NVU

### The NVU: regulation of TJ assembly by perivascular cells

#### Developmental role of astrocyte and pericyte secreted proteins

Development and maintenance of the BBB requires functional interactions between endothelial cells and perivascular cells of the NVU: whereas astrocytes have been well documented to regulate BBB formation and integrity [76,77], only recently was the role of pericytes unraveled (for reviews: [78-80]).

**Figure 1:**
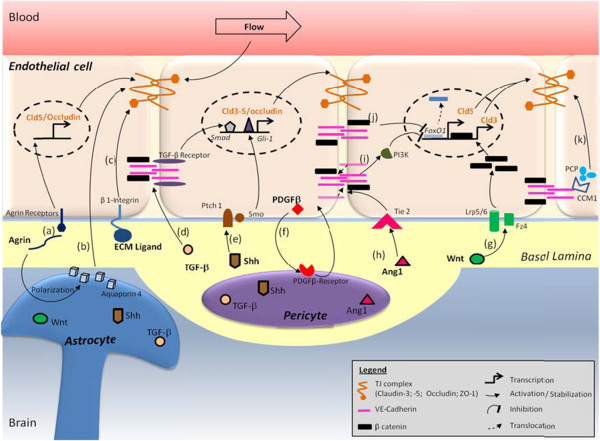
**Schematic representation of TJ modulation by the NVU.** (**a**) The basal lamina protein agrin increases claudin-5 (Cld5) and occludin expression [92]; (**b**) Aquaporin-4 density, regulated by agrin, stabilizes TJ complexes through ZO-1 expression [93]; (**c**) β1-integrin engagement stabilizes Cld5 localization at the TJ [94]; (**d**) astrocyte/pericyte-secreted TGF-β induces Cld5 transcription through activation of Smad transcription factor [91]; (**e**) Shh enhances expression of TJ proteins via its membrane receptor Ptch1/Smo and the transcription factor Gli-1 [95]; (**f**) Endothelial PDGF-β recruits pericytes which stabilize BBB phenotype [90]; (**g**) Wnt 7a/7b proteins, via their membrane receptors Frizzled-4 associated to LRP5/6, induce Cld3 transcription through stabilization of β-catenin [96]; (**hij**) Angiopoietin-1 (Ang1), via its membrane receptor Tie2, enhances VE-Cadherin clustering and Cld5 transcription through inhibition of FoxO1 activity by PI3K and β-catenin sequestration [83,97] ; (**k**) VE-Cadherin engagement recruits CCM1 and the polarity complex (PCP) leading to TJ stabilization [98] .

Indeed, early studies using co-culture of cerebral endothelial cells and astrocytes (or culture in the presence of astrocyte-conditioned medium) [81-87] highlighted the role of astrocyte-derived soluble factors in maintaining the specialized phenotype of brain endothelial cells (Figure 1). In addition, more recent reports established that pericytes also actively contribute to BBB formation during development by the release of several growth factors and morphogens [88-91].

Astrocyte- and pericyte-derived Wnt and hedgehog morphogens were reported to control BBB formation during development and TJ integrity. Indeed, the Wnt/β-catenin pathway has been recently discovered as a major BBB-regulating pathway. Wnt ligation to its membrane receptors, Frizzled4 (Fz4) and LRP5/6 expressed by brain endothelial cells, inhibits the β-catenin repressor complex, allowing β-catenin cytoplasmic accumulation, nuclear translocation and transcription of various genes, including claudin-3 in cultured murine brain endothelial cells [96,99] (Figure 1). Moreover, *in vivo* inactivation of Wnt factors (Wnt7a and Wnt7b) [100], Fz4 receptor [101] or injection of a soluble inhibitor of the Wnt/ Frizzled receptor interaction [102] lead to major vascular defects in the CNS (interestingly, *not* in non-neuronal tissues) and to BBB breakdown, clearly demonstrating a specific role for the Wnt/β-catenin pathway in BBB differentiation during development and for BBB maintenance in adulthood. These exciting observations (for review, see: [103]) open new research avenues for controlling BBB permeability in pathological situations as well as improving drug delivery to the CNS.

Sonic hedgehog (Shh), another well-known morphogen protein, acting through its membrane receptors Patched-1 (Ptch1)/Smoothened (Smo), was also recently shown to control BBB differentiation and to maintain the immune privilege of the CNS by inhibiting the endothelial production of chemokines and expression of adhesion proteins supporting extravasation of leukocytes to the brain [95].

In conclusion, these recent findings further document, at the molecular and cellular levels, the functional interactions between brain endothelial cells, pericytes and astrocytes and emphasise the key importance of the NVU in controlling BBB permeability and integrity. The major cellular cross-talks at the NVU are illustrated in Figure 1.

#### Role of basement membrane-associated proteins

The vascular basement membrane (or basal lamina) is a complex structure, composed of four glycoprotein families: laminins, collagen type IV, nidogens and heparan sulfate proteoglycans. Recent studies have unraveled the contribution of the endothelial laminin isoform α5 to the barrier property of the BBB by selectively inhibiting lymphocyte infiltration; the basement membrane thus contributes to maintain the well-known “immune privilege” of the CNS [104].

The heparan sulfate proteoglycan agrin is found in the basal lamina of brain microvessels [105]. A strict positive correlation has been reported between agrin deposition and expression of occludin [106], whereas, conversely, absence of agrin in glioblastoma vessels was shown to correlate with the lack of TJ proteins (occludin, claudin-5): these observations strongly suggest that agrin may regulate TJ formation in brain endothelium. Recently, agrin was described to be involved in the development of the BBB by contributing to astrocyte polarity [92]. Moreover, β1-integrin-mediated attachment of brain endothelial cells to the basement membrane has also been reported to be critical for stabilizing claudin-5 localization at TJs and maintaining BBB integrity *in vitro* and *in vivo*[93]. Genetic deletion of β1-integrin decreases the expression of the polarity protein Par-3, leading to the loss of endothelial cell polarity: these recent data suggest that β1-integrin-mediated brain endothelial cell adhesion to the basement membrane may lead to the development of cell polarity, TJ formation and BBB integrity [94].

### VE-cadherin and β-catenin as modulators of TJs

In addition to TJs, junctional complexes between endothelial cells include adherens junctions (AJs), constituted by transmembrane proteins VE-cadherin linked to the actin cytoskeleton through catenins (*eg*: p120-catenin, α- and β-catenin) [107-109]. Interestingly, AJ and TJ complexes functionally interact in brain endothelial cells: indeed, VE-cadherin engagement induces claudin-5 transcription through inhibition of FoxO1 activity (a transcription repressor of claudin-5 gene) and β-catenin sequestration (a stabilizer of FoxO1 activity) in AJ complexes [97], in line with the above-mentioned capacity of β-catenin, downstream of Wnt receptor activation, to control claudin gene expression [96]. These findings clearly place VE-cadherin upstream of claudin-5 in the establishment, maturation and maintenance of endothelial cell-cell junctions.

### Contribution of shear stress to TJ modulation and BBB integrity

It is established that one important mechanical stimulus contributing to BBB formation and maintenance is shear stress [110], a tangential force generated by flow across the apical surface of vascular endothelium [111,112]. In line with the accepted concept that cerebral microcirculation is highly heterogeneous, mean shear stress levels in brain microvessels has been estimated in a range as wide as 0.01 to 10 dynes/cm^2^ in capillaries and 10–100 dynes/cm^2^ in arterioles [113-115]. Several dynamic *in vitro* models were developed in order to mimic a physiological situation (using laminar, steady flow) or a pathological condition (such as atherosclerosis), using an irregular flow. Interestingly, culturing human umbilical vein endothelial cells (HUVECs) in a laminar flow chamber in the presence of meningococci (*N*. *meningitidis*) was instrumental for unraveling some key molecular mechanisms of CNS invasion by these meningitis-causing human pathogens [111]. Regarding BBB differentiation, culture of brain endothelial cells under flow has been reported to induce the expression of the TJ proteins occludin and ZO-1, to promote actin cytoskeleton reorganization and to reduce endothelial permeability [113,115-117]. In addition, very recent findings suggest that physiological shear stress (6 dynes/cm^2^) may increase the expression of a variety of BBB-associated genes in human brain microvascular endothelial cells, such as genes encoding for TJ proteins (ZO1, claudin-3, claudin-5), several influx transporters (Glut-1) and multidrug resistance efflux transporters (ABCB1/P-gp, ABCC5/MRP5) [116]. Nevertheless, further investigation is still required to get a better understanding of the contribution of shear stress to the maintenance of BBB integrity.

## Dysregulation of the BBB via phosphorylation and relocalization of TJ proteins

Studies on CNS diseases associated with BBB dysfunctions (e.g. stroke, multiple sclerosis, cerebral infection, brain tumors, Parkinson’s and Alzheimer’s diseases) have pointed to various molecular mechanisms involved in disruption of TJ integrity, notably including Serine/Threonine (Ser/Thr)- and Tyrosine (Tyr)-phosphorylation, down-regulation, degradation or translocation of TJ proteins; a non exhaustive list of related reports are presented in Table 1. More than other TJ proteins (such as claudins or JAMs), occludin has been the focus of numerous studies investigating post-translational modifications and their consequences on TJ integrity (see for review: [118,119]).

**Table 1:** **Dysregulation of the BBB via phosphorylation or down**-**regulation of TJ proteins**

**TJ proteins modifications**	**Targeted TJ proteins**	**Signaling pathway**	**Stimulus** / **Diseases**	**References**
**Serine**/**Threonine Phosphorylation**	Cld5 (Thr207)	PKA	cAMP	[120]
	Cld5, Occludin and ZO-1	nPKC-θ / aPKC-ζ	Hypoxia	[121]
	N.D.	cPKC-α, cPKC-βII , aPKC-λ/ζ	HIV-1 gp120	[122]
	Cld5 (T207) Occludin (T382/S507)	RhoA/Rho kinase	HIV-1 encephalitis	[123]
	Cld5, Occludin, ZO-1	RhoA / PKC-α	CCL2 chemokine	[124]
	Cld5 and Occludin	MLCK	Alcohol / Reactive oxygen species	[125,126]
	N.D.		Hypoxia / Reactive oxygen species	[127]
**Tyrosine Phosphorylation**	Occludin	c-Src	Cerebral ischemia	[128]
	Occludin	N.D.	Glutamate	[129]
	Cld5	N.D.	TGF-β	[130]
	ZO-1	ND	Tyrosine phosphatase inhibition	[131]
**Down**-**regulation or degradation**	Cld5 and Occludin Internalization	Caveolae-dependent endocytosis	CCL2 chemokine	[132]
	Occludin	JNK, p38MAPK	Amyloid-β peptide	[133]
	Cld5	ERK1/2	HIV-1 Tat protein	[134]
	Occludin and ZO-1 distribution	PLC-γ, PI3K/Akt	Hypoxia	[135]
		N.D.		[136]
	Cld5	N.D.		[137]
	Cld5 and Occludin	VEGFR	VEGF	[138]
	Cld3	N.D.	Multiple Sclerosis Glioblastoma multiforme	[41]
	Cld5, Occludin, ZO-1	nPKC-δ	Cerebral ischemia	[139]
	Occludin and ZO-1	MLCK	HTLV-1	[140]
	Cld5 and Occludin	RhoA/RhoK	Reactive oxygen species	[141]

N.D : Not Described.

### Ser/Thr-phosphorylation of TJ proteins and regulation of barrier permeability

Ser/Thr-phosphorylation forms of occludin are found concentrated at TJs whereas dephosphorylated occludin is rather detected on basolateral membranes and associated with disrupted TJs in epithelial cells [142,143] as well as in brain endothelial cells in experimental autoimmune encephalomyelitis, a murine model of multiple sclerosis characterized by brain inflammation [144]. Regarding claudin-5, phosphorylation of its C-terminal domain on Thr207 residue in response to PKA or Rho kinase activation [120,123,145] generally affected TJ integrity in brain endothelial cells and increased permeability.

#### Differential regulation of TJs by Protein Kinases C (PKCs)

PKC-dependent pathways have been involved in endothelial barrier disruption, as reported following treatment by pertussis toxin, an inhibitor of Gαi heterotrimeric G proteins [146], or in response to the pro-inflammatory cytokine interleukin-6 (IL-6) which plays a critical role during hypoxia [147]. However, early reports had clearly established that PKC activity was crucial for BBB integrity in epithelial cells, inasmuch as PKC inhibitors completely blocked the formation of TJs [148,149]; in addition, PKC-mediated phosphorylation of occludin (on residue Ser338) was involved in occludin targeting to TJs and TJ stabilization in epithelial MDCK cells [148].

At least part of the interpretation of these apparently conflicting data may be found in the heterogeneity of the PKC family. The Ser/Thr-kinases PKCs are indeed classified into conventional (cPKC: α, βI, βII and γ), novel (nPKC: δ, ε, θ, η, μ) and atypical (aPKC: λ,ζ) PKC isozymes [150] according to their modes of regulation. Accumulating evidence has pointed to a differential capacity of PKC isozymes to regulate BBB permeability. Indeed, activation of nPKC-θ and aPKC-ζ signaling by hypoxia-mediated TJ proteins results in relocalization (such as claudin-5, occludin, ZO-1) and increased BBB permeability in rat brain microvascular endothelial cells (*in vitro* and *in vivo*) [121,151]. In human brain microvascular endothelial cells, cPKCα, cPKC βII and aPKCλ/ζ isoforms were activated by HIV-1 gp120 envelope protein, leading to BBB disruption, intracellular calcium increase and monocyte migration across cell monolayer [122]. Interestingly, when cPKC-α was found to contribute to TJ disassembly, nPKC-ε activation mediated TJ formation in epithelial MDCK cells [152]. In line with this observation, over-expression of cPKC-α in rat epididymal microvascular endothelial cells was reported to enhance thrombin-induced permeability, whereas nPKC-δ expression promoted barrier function [153]. By contrast, IL-25, expressed by mouse brain capillary endothelial cells, was shown to prevent inflammation-induced BBB disruption and down-regulation of TJ proteins (occludin, claudin-5, JAMs) through activation of the nPKC-ε pathway [154]. Altogether, these observations strongly suggest that nPKC-selective activation generally contributes to maintaining barrier integrity, whereas cPKC activation has the opposite effect, both in polarized epithelium and endothelium (Table 1).

Regarding aPKC isoforms (λ and ζ), they have been shown to contribute to the establishment of epithelial cell polarity, via participation in the PCP complex together with Par-3 and Par-6 [63,68,155]. As mentioned above, the PCP complex is recruited to endothelial TJs by Par-3 binding to JAM proteins [64-66]. Over-expression of a dominant negative mutant of aPKC causes mislocalization of Par-3 and affects the biogenesis of the TJs in epithelial cells [67], suggesting that Par-3 is a substrate of aPKC and that its localization in epithelial cells is dependent upon its phosphorylation. In the same line, the VE-cadherin/CCM1 (a protein encoded by the CCM1 gene which is mutated in a large proportion of patients affected by cerebral cavernous malformation) complex controls aPKC-ζ activation and Par-3 localization during early steps of brain endothelial cell polarization [98]. The participation of this PCP complex to TJ integrity was further illustrated by the recent observation that meningococcal adhesion to human cerebral endothelial cells recruited Par-3, Par-6 and aPKC-ζ under bacterial colonies and induced disruption of cell-cell junctions [156]. Surprisingly, a distinct Par-3/Par-6 complex, directly associated with VE-cadherin and lacking aPKC, has also been identified in endothelial cells [157]. Finally, although additional polarity complexes are known in epithelial cells (the apical Crumbs complex and the basolateral Scribble complex) where they also contribute to TJ formation and regulation, no similar observations have been reported, to our knowledge, in brain endothelial cells.

#### BBB disruption mediated by Rho/ Rho kinase and MLCK activation

The RhoA GTPase signaling pathway, activated by several membrane receptors, has been extensively documented in various cell types to induce actin cytoskeleton rearrangements involved in cell migration and proliferation. In brain endothelial cells, RhoA activation increased permeability, in response to inflammatory stimuli, through one of its major effectors Rho kinase (ROCK) [158,159]. Among these inflammatory stimuli, chemokines like MCP-1/CCL2, acting via their seven transmembrane-domain receptors, are known to activate the RhoA/ROCK pathway in mouse brain endothelial cells, to induce occludin, claudin-5 and ZO-1 Ser/Thr-phosphorylation, followed by their delocalization from TJs, ultimately leading to increased barrier permeability [124,160]. Similarly, enhanced monocyte migration across human brain endothelial cells was observed in an HIV-1 encephalitis model [123,161]. Also, adhesion molecules like ICAM-1 and VCAM-1 were shown, in response to lymphocyte/monocyte adhesion, to transduce signals in rat brain endothelial cell lines including activation of the RhoA/ ROCK pathway [162,163]: activation of this pathway ultimately leads to enhanced lymphocyte migration, suggesting that this process may be involved in the massive infiltration of immune cells into the CNS observed in multiple sclerosis. It must be mentioned, however, that lymphocyte migration across the BBB may also happen via a transcellular pathway, leaving intact endothelial TJs [164].

Rearrangements of the actin cytoskeleton have long been recognized to be regulated, not only by the RhoA/ROCK pathway, but also, often in a coordinated manner, by the myosin light chain kinase (MLCK): MLCK directly phosphorylates the myosin light chain, leading to actomyosin contraction and endothelial barrier disruption [165-167]. In the same line, inhibition of MLCK in bovine brain endothelial cells was more recently reported to prevent hypoxia-induced BBB disruption [127], whereas alcohol increased human brain endothelial cell permeability via activation of MLCK and phosphorylation of occludin and claudin-5 [125,126]. Recently, pro-inflammatory cytokines (IL1β and TNFα), secreted by lymphocytes chronically infected by the HTLV-1 retrovirus, were reported to induce barrier disruption in the human brain endothelial cell line hCMEC/D3, associated with loss of occludin and ZO-1 through activation of the MLCK pathway [140].

In conclusion, as summarized in Table 1, inflammation- or infection-induced actin cytoskeleton rearrangements in brain endothelial cells, mediated by the RhoA/ROCK and/or MLCK pathways, are associated with the phosphorylation, followed by delocalization or degradation of TJ proteins, and BBB disruption.

### BBB dysregulation by Tyr-phosphorylation of TJ proteins

Early studies with cultured bovine brain endothelial cells and MDCK cells had pointed to Tyr-phosphorylation as a mechanism for increasing TJ permeability [131]. Accumulating evidence demonstrated that Tyr-phosphorylation of TJ proteins, as well as AJ proteins, was directly involved in BBB disruption, as observed in various pathological situations, although the identity of the Tyr-kinases involved often remained unknown. Unlike occludin Ser/Thr phosphorylation associated with barrier formation, as mentioned above, occludin Tyr-phosphorylation was reported to be associated with increased permeability of cultured rat brain endothelial cells exposed to glutamate, as a way to mimic cerebral ischemia [129] (Table 1). Like other pro-inflammatory cytokines, transforming growth factor (TGF)-β1 is known to increase BBB permeability: as recently reported in bovine retinal and human brain endothelial cells, this effect was mediated by Tyr-phosphorylation of both claudin-5 and VE-cadherin [130]. Vascular endothelial growth factor (VEGF), a major angiogenic factor, which is drastically enhanced in response to hypoxia, promotes Tyr-phosphorylation of TJ proteins (ZO-1, occludin) in mouse brain and retinal endothelial cells [168,169] either directly via its membrane receptor tyrosine kinase VEGFR2 or via the activation of the cytosolic tyrosine kinase c-src [128,170]. VEGF-mediated Tyr-phosphorylation of TJ proteins in brain endothelial cells was often followed by their down-regulation and/or re-localization, leading to TJ destabilization and permeability increase [136-138,171].

### Alterations of expression and localization of TJ proteins

Caveolae are specialized plasma membrane microdomains, abundantly found in endothelial cells where they mediate various biological events such as transcytosis, vascular permeability and angiogenesis [172,173]. They are enriched in the small membrane protein caveolin-1 which has been shown to recruit TJ proteins [9,174]. Caveolae-mediated endocytosis induced by actin depolymerization was reported to evoke occludin internalization in MDCK cells [175]. Interestingly, exposure of cultured rat brain endothelial cells to the HIV-1 Tat protein was reported to increase TJ permeability, through alterations in expression and distribution of TJs proteins: occludin, claudin-5, ZO1, ZO2 [134,176]. In the same line, the increase in TJ permeability observed in mouse brain endothelial cell response to the inflammatory cytokine CCL2 was recently shown to be associated with claudin-5 and occludin internalization in a caveolae-dependent manner [132]. Altogether these results strongly support the conclusion that alterations in expression and localization of TJ proteins, associated or not with their phosphorylation in response to various pathological stimuli, directly contribute to TJ disruption and BBB permeability increase (Table 1); in addition, they suggest a role of caveolin-1/caveolae in such TJ remodeling.

## Conclusion

The brain endothelial TJ complex, which constitutes a key feature of the BBB, is now understood as a scaffolding and signaling platform in close interaction with the actin cytoskeleton and the AJ complex. It also appears as a dynamic complex, submitted to post-translational modifications in response to physiological and pathological stimuli. Indeed, perivascular cells of the NVU, notably astrocytes and pericytes, secrete multiple growth factors and morphogens that contribute to TJ formation and integrity. Conversely, various pathological situations associated with the presence of inflammatory cytokines, reactive oxygen species or pathogens, lead to TJ disruption following phosphorylation and/or internalization of TJ proteins.

Although our understanding of TJ architecture and function has significantly increased over the last ten years, a number of issues will have to be addressed in the next future, in particular taking advantage of new and/or global analysis technologies. For example, super-resolution light microscopy (time-lapse stimulated emission depletion (STED) imaging) recently appeared as a very powerful approach to unravel synapse assembly and plasticity [177]; in the same line, super-resolution microscopy of TJs (with a resolution down to 50–80 nm) of cerebral microvessels in brain slices should provide a more accurate understanding of TJ organization and dynamics. Also, thanks to the availability of validated BBB *in vitro* models, identification by mass spectrometry (MS/MS analysis) of the secreted proteins (so-called ‘secretome’) from brain endothelial co-cultures with astrocytes or pericytes may unravel new paracrine signaling pathways in the NVU which contribute to the stabilization of TJs at the BBB; in addition, similar analyses in the presence of inflammatory agents or pathogens [178] may highlight unsuspected mechanisms of TJ disruption. This approach will complement quantitative targeted absolute proteomics (also known as selected reaction monitoring (SRM)), an emerging approach to quantify membrane proteins [179]. This technology will also greatly benefit the field, allowing absolute quantification of TJ proteins in physiological and various pharmacological situations, as recently proposed [180]. The treatment of neurological diseases is currently hampered by difficulties encountered in delivering therapeutic compounds to the brain, across the BBB. Because previous drug delivery strategies based on transcellular transport machinery have shown limited efficacy so far, it is tempting to propose that transient modulation of TJs at the BBB, using *in vitro* models of the BBB and *in vivo* models of human pathologies, may constitute an alternative approach for drug delivery to the brain. Clearly, this field will benefit greatly from an in-depth understanding of TJ architecture and functional regulatory mechanisms.

## Abbreviations

AJ: Adherens junction; BBB: Blood brain barrier; CNS: Central nervous system; ECL: Extracellular loop; Fz: Frizzled; HUVEC: Human umbilical vein endothelial cell; IL: Interleukin; JAM: Junctional adhesion molecule; MAGUK: Membrane associated guanylate kinase; MLCK: Myosin light chain kinase; NVU: Neurovascular unit; PCP: Planar cell polarity; PKC: Protein kinase C; Ptch1: Patched-1; ROCK: Rho kinase; Shh: Sonic hedgehog; Smo: Smoothened; TAMP: Tight junction-associated marvel proteins; TGF: Transforming growth factor; TJ: Tight junction; VEGF: Vascular endothelial growth factor; ZO: Zonula occludens.

## Competing interests

The authors declare no conflict of interest.

## Authors’ contributions

ACL and CA: were responsible for the collection of data and references, and for the drafting of the document. FG and KG were involved in the collection of data and drafting of the document. POC was responsible for the drafting and editing of the document, and for the discussion of the data. All authors read and approved the final manuscript.
